# 90. The Diagnosis of Ventilator-associated pneumonia (DIVA) trial: Results of a Clinical Trial of a Bundled VAP Diagnostic Stewardship Intervention

**DOI:** 10.1093/ofid/ofad500.006

**Published:** 2023-11-27

**Authors:** Owen Albin, Jonathan Troost, Michael Thomas, Robert Hyzy, Mark Konkle, Weirauch J Andrew, Krishna Rao, Keith S Kaye

**Affiliations:** University of Michigan Medical School, Ann Arbor, MI; University of Michigan Medical School, Ann Arbor, MI; University of Michigan Medical School, Ann Arbor, MI; University of Michigan Medical School, Ann Arbor, MI; Michigan Medicine, Ann Arbor, Michigan; Michigan Medicine, Ann Arbor, Michigan; Department of Internal Medicine, Infectious Diseases Division University of Michigan, Ann Arbor, Michigan, Ann Arbor, MI; Rutgers Robert Wood Johnson Medical School

## Abstract

**Background:**

Ventilator-associated pneumonia (VAP) is commonly overdiagnosed & a primary driver of antibiotic overuse within intensive care units (ICUs). Antimicrobial stewardship programs have successfully leveraged diagnostic stewardship interventions (DSIs) to prevent overdiagnosis/overtreatment of diverse clinical syndromes, yet this approach has not been extended to VAP. In this trial, we aimed to evaluate the safety, feasibility & efficacy of a novel DSI care bundle (DSI-CB) targeting VAP.

**Methods:**

The DIVA trial (NCT05176353) was a pilot/feasibility trial conducted in 2 ICUs at Michigan Medicine from February 2022-February 2023. A DSI-CB targeting the respiratory culture testing pathway was implemented sequentially in study ICUs, using an interruptive electronic health record clinical decision support tool & modifications to microbiology laboratory workflows (Table 1). Providers were counseled on DSI-CB use during bimonthly educational sessions during trial rollout & via monthly email reminders thereafter. Rates of prespecified co-primary safety and secondary efficacy outcomes were compared between the postintervention study cohort & 5-year preintervention historical controls using negative binomial regression. Interrupted time series analysis was used to evaluate ICU antibiotic utilization rates (ICU-AURs) to account for temporal trends.

**Figure:**
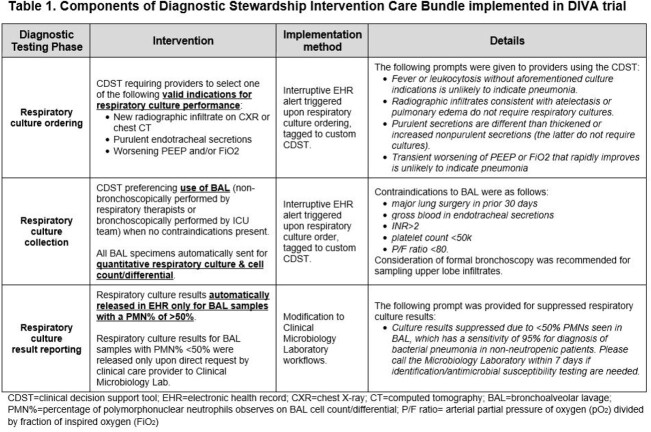

**Results:**

1810 patients were admitted to study ICUs following DSI-CB implementation, 29% of whom were eligible for DSI-CB use. DSI-CB was utilized in 77% of eligible patients. Patient demographics, comorbidities & measures of acute severity of illness were similar pre- & post-intervention. DSI-CB implementation was not associated with increases in primary adverse safety outcomes (Table 2). DSI-CB implementation was associated with significant reductions in rates of total respiratory cultures ordered, rates of positive respiratory cultures & reductions in both total & broad-spectrum ICU-AURs (Table 2, Figure 1).

**Figure:**
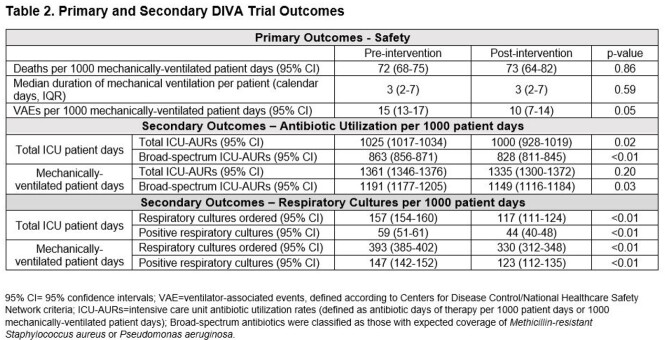

**Figure:**
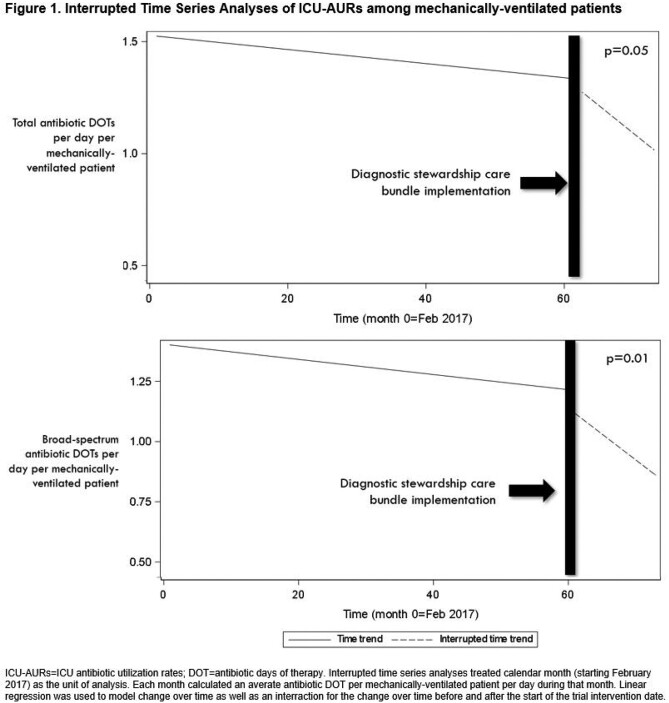

**Conclusion:**

Implementation of a novel VAP DSI-CB was safe, feasible & associated with significant reductions in rates of respiratory culture positivity and ICU-AURs. This represents the first trial of a DSI targeting VAP & a novel avenue for ICU antimicrobial stewardship. Large-scale trials are warranted.

**Disclosures:**

**Owen Albin, MD**, Biomeriux: Grant/Research Support|Charles River Laboratories: Advisor/Consultant|Shionogi: Advisor/Consultant **Weirauch J. Andrew, RRT**, Drager Medical: Equipment loaned for research purposes, but no monetary funds were provided.|Jones and Bartlett Learning: Honoraria **Krishna Rao, MD, MS**, Merck & Co.: Grant/Research Support|Rebiotix: Advisor/Consultant|Seres Therapeutics: Advisor/Consultant|Summit Therapeutics: Advisor/Consultant **Keith S. Kaye, MD, MPH**, Entasis: Advisor/Consultant|Entasis: Honoraria|GSK: Advisor/Consultant|GSK: Honoraria|Merck: Advisor/Consultant|Merck: Honoraria|Shionogi: Advisor/Consultant|Shionogi: Honoraria|VenatoRx: Advisor/Consultant|VenatoRx: Honoraria

